# Collagen type IV alpha 1 (COL4A1) silence hampers the invasion, migration and epithelial–mesenchymal transition (EMT) of gastric cancer cells through blocking Hedgehog signaling pathway

**DOI:** 10.1080/21655979.2022.2053799

**Published:** 2022-03-29

**Authors:** Xijuan Cui, Tao Shan, Lina Qiao

**Affiliations:** Department of General Surgery, The First Affiliated Hospital of Xi’an Jiaotong University, Xi ‘An, P.R. China

**Keywords:** Gastric cancer, COL4A1, migration, invasion, EMT, Hedgehog signaling pathway

## Abstract

Gastric cancer (GC), which features high prevalence and mortality rate, remains the third most lethal cancer worldwide. The paper was designed to explore the impacts of collagen type IV alpha 1 (COL4A1) on GC, along with its potential mechanism. The mRNA and protein expressions of COL4A1 in GC cells were assessed using RT-qPCR and Western blot. After depleting COL4A1, RT-qPCR and Western blot were conducted again to check the transfection efficacy. With the application of CCK-8, wound healing and transwell, the capabilities of cells to proliferate, migrate and invade were appraised, respectively. Moreover, Western blot tested the protein levels of factors involved in migration, proliferation, epithelial–mesenchymal transition (EMT) and Hedgehog signaling. As a result, COL4A1 displayed elevated expression in GC tissues and cells, while its knockdown inhibited the cell viability, migration, invasion and EMT in GC. According to Gene Set Enrichment Analysis (GSEA), COL4A1 was involved in the regulation of Hedgehog signaling pathway, which was then further verified by the detection of Hedgehog-related proteins. To figure out the relationship between COL4A1 and Hedgehog signaling pathway, we used purmorphamine, an agonist of Hedgehog, to treat GC cells, finding that COL4A1 blocked Hedgehog signaling to inhibit the aggressive phenotypes of GC cells. In short, COL4A1 silence was testified to exhibit suppressive effects on the malignant process of GC, suggesting that COL4A1 might be a potent hallmark of GC therapy.

## Graphical Abstract

**Figure:**
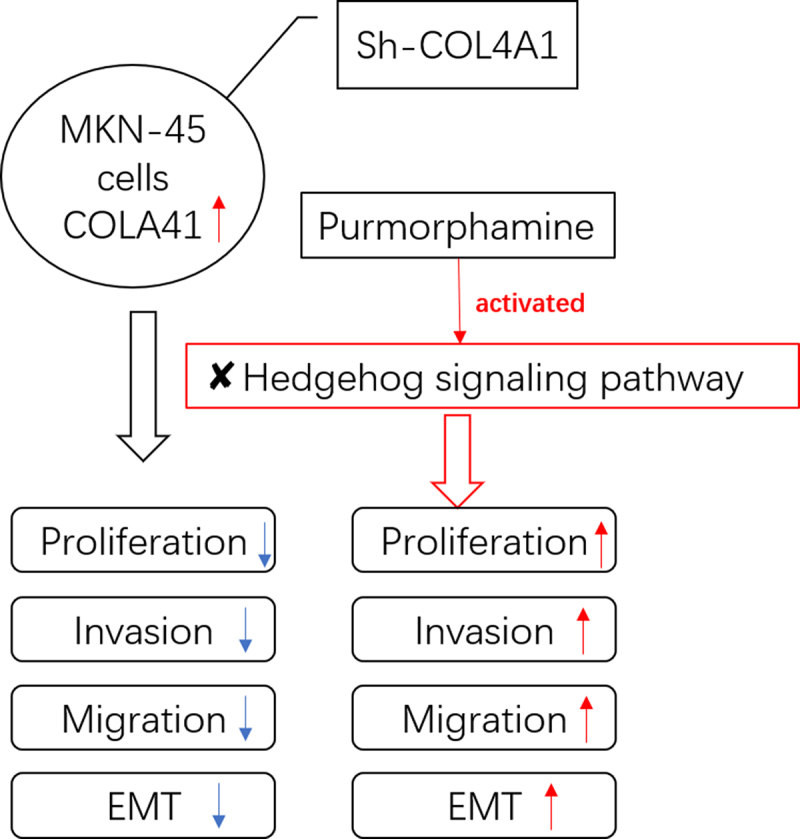

## Introduction

Gastric cancer (GC) is a major health burden globally and features a high incidence and mortality rate [1,2]. It was reported that about 880,000 GC cases were diagnosed and 650,000 patients living with GC were dead in 2000 [3]. As a multifactorial disease, GC results from many risk factors, including environment, age, sex, smoking, gene and *Helicobacter pylori*, among which *H. pylori* was viewed as the primary cause [4,5]. Nowadays, surgery, chemotherapy and radiation therapy are the dominant therapeutic strategies for GC [6]. Despite the fact that surgical resection has better efficacy for GC and chemotherapy improves the overall survival rate, the prognosis of patients suffering from GC still remains poor and the 5‐year survival rate is only about 20% for patients with advanced GC [7]. In this way, the early diagnosis as well as effective therapeutic methods for GC is of great importance to enhance the survival rate of GC patients.

Collagens are abundant in extracellular matrix (ECM), which acts as an important player in tumor microenvironment and the regulation of tumor cell behaviors [8,9]. As the most abundant component of basement membranes of extracellular matrix [10], collagen IV is primarily generated and secreted from fibroblasts, endothelial cells and epithelial cells [11,12]. Being a collagen IV molecule, collagen type IV alpha 1 (COL4A1) participates in the interactions among cells and owns two specific recognition sites for integrins alpha 1 beta 1 and alpha 2 beta 1 [13]. Studies in growing numbers have testified that the upregulated COL4A1 promoted the migration, invasion and proliferation of cancers, such as breast cancer and bladder cancer [14,15]. Although previous studies have identified COL4A1 as a key gene in the development, progression and recurrence of gastric cancer and testified that COL4A1 was concerned with its poor prognosis [16–18], the molecular mechanism of COL4A1 in gastric cancer progression has not been elucidated.

The Hedgehog signaling pathway acts as an indispensable player in the development of invertebrates and vertebrates [19]. It was reported that the activation of Hedgehog signaling pathway is involved in the progression and metastasis of many malignant tumors [20], including ovarian cancer [21] and pancreatic cancer [22]; therefore, the inhibition of Hedgehog signaling pathway was considered to be an effective intervention for the treatment of human cancer.

The aim of this study was to investigate the role of COL4A1 in GC cells. We hypothesized that COL4A1 silence could suppress the invasion, migration and EMT of GC cells through blocking Hedgehog signaling pathway. One kind of GC cells (MKN-45 cells) was selected, and short hairpin RNAs (sh-RNA) specific to COL4A1 (sh-COL4A1) were transfected into MKN-45 cells to see the effects of COL4A1 silence on cell invasion, migration, EMT and Hedgehog signaling pathway. An agonist of Hedgehog signaling pathway was used to reversely validate that COL4A1 silence could block Hedgehog signaling pathway.

## Material and methods

### Cell culture, treatment and transfection

GC cell lines, including AGS, MKN74, HGC-27, MKN-45 and human gastric epithelium (GES-1) cell line, were provided by the American Type Culture Collection (ATCC). 10% fetal bovine serum (FBS, Clark Bioscience) was added to RPMI-1640 medium (Corning, Corning, NY) to incubate cells at 37°C with 5% CO_2_. Subsequently, purmorphamine, an agonist of Hedgehog signaling pathway, was used to treat transfected cells.

sh-COL4A1#1, sh-COL4A1#2 and its corresponding negative control (shRNA-NC) were procured from GenScript Biotech. With the adoption of Lipofectamine 2000 transfection reagent (Invitrogen), the transfection was carried out.

## Reverse transcription-quantitative polymerase chain reaction (RT-qPCR)

Total RNA that lysed by TRIzol reagent (Takara, Osaka, Japan) was then synthesized into complementary DNA (cDNA). Real-time PCR for gene quantitation assay was performed on ABI 7500 quantitative PCR instrument (ABI/Perkin Elmer) with the help of SYBR-Green Supermix (Invitrogen). Finally, the relative gene expressions were determined by 2^−ΔΔCt^ method. The primers used in this study were displayed as follows: COL4A1 forward primer: 5’- GGACTACCTGGAACAAAAGGG-3’ and reverse primer: 5’-GCCAAGTATCTCACCTGGATCA-3’.

## Western blot

The proteins isolated by RIPA lysis buffer (Solarbio) were quantified with a bicinchoninic acid (BCA) protein assay kit (Thermo Fisher Scientific Inc.). After being exposed to 12% gel with SDS–PAGE, PVDF membranes were utilized for protein transfer. Inhibited with 5% nonfat milk, the membranes were subsequently cultivated with primary antibodies against COL4A1 (#50,273; 1:1000; Cell signaling pathway), Ki67 (ab92742; 1:5000; Abcam), PCNA (ab92552; 1:1000; Abcam), MMP2 (ab92536; 1:2000; Abcam), MMP9 (ab76003; 1:2000; Abcam), N-cadherin (ab76011; 1:5000; Abcam), vimentin (ab92547; 1:2000; Abcam), E-cadherin (#3195; 1:1000; Cell signaling pathway), Gli1 (ab134906; 1:500; Abcam), Shh (#2207; 1:1000; Cell signaling pathway) and GAPDH (ab9485; 1:2500; Abcam) at 4°C overnight. The secondary antibodies were then adopted to incubate the membranes on the next day. Finally, the visualization of blots was captured through enhanced chemiluminescence (ECL) reagents.

## Cell counting kit-8 (CCK-8)

96-well plate was taken to inoculate MKN-45 cells. After 24, 48 and 72 h, 10% CCK-8 solution was supplemented into each well to maintain the cells for another 2 h. Eventually, plates at 450 nm were read using a microplate reader (BioTek).

## Wound healing

MKN-45 cells were inoculated into 6-well plates and cultured until the cells have reached 90% confluence. Scratched with sterile pipette tips, a wound across the cell surface was made. Following the rinse with PBS three times, the cells were incubated at 37°C with 5% CO_2_ and recorded at 0 and 24 h. Finally, the relative migration rate was observed by Image-J software.

## Transwell

In brief, MKN-45 cells were plated in the transwell upper chambers, and the lower well was supplemented with a medium containing 10% FBS. 24 h later, the fixation and staining of MKN-45 cells were conducted with 4% paraformaldehyde and 0.1% crystal violet, individually. Finally, the images of invaded cells were photographed by a microscope.

## Statistical analysis

Data obtained from the experiments were displayed as mean ± standard deviation and analyzed with GraphPad Prism software (version 8.0; La Jolla, CA, USA). One-way ANOVA as well as Tukey’s test was used to analyze differences among different groups. It was deemed to be statistically significant at *P* < 0.05.

## Results

### COL4A1 possessed high expression in GC

**Figure 1.**
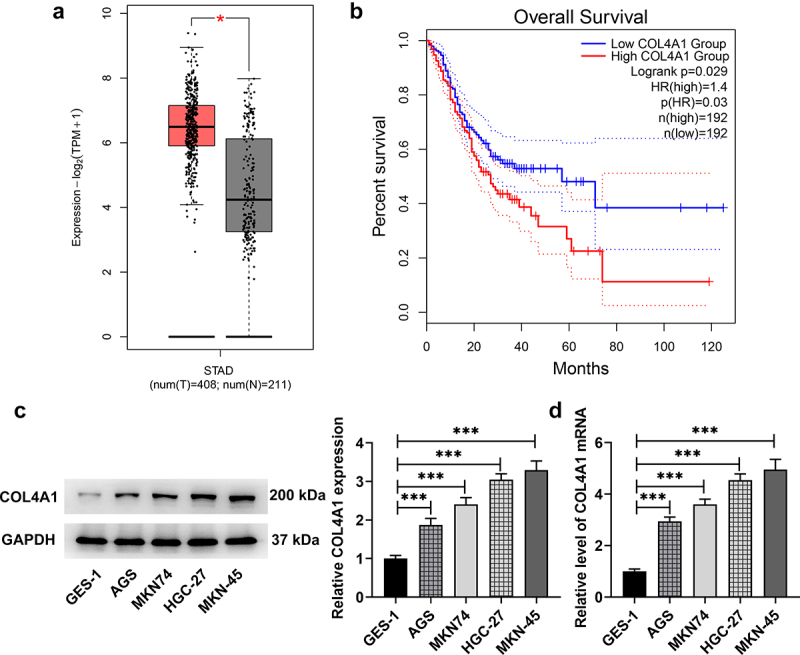
COL4A1 possessed high expression in GC. (a,b) GEPIA2 examined COL4A1 expression in GC tissues and the correlation between COL4A1 and the prognosis of GC. (c,d) RT-qPCR and Western blot checked COL4A1 expression in GC cells.

Data on GEPIA2 database (http://gepia.cancer-pku.cn/) revealed that COL4A1 was upregulated in GC tumor tissues and had a close relation with the prognosis of GC (Figure1a-b). In this study, the expression of COL4A1 in GC cells was further measured. As Figure 1c-d demonstrates, COL4A1 gained a huge growth in GC cells (AGS, MKN74, HGC-27 and MKN-45) at both mRNA and protein levels, especially in MKN-45 cells in comparison with that in normal cell line (GES-1); in view of this, we chose MKN-45 cells for the following experiments.

## COL4A1 silence abrogated MKN-45 cell proliferation

**Figure 2.**
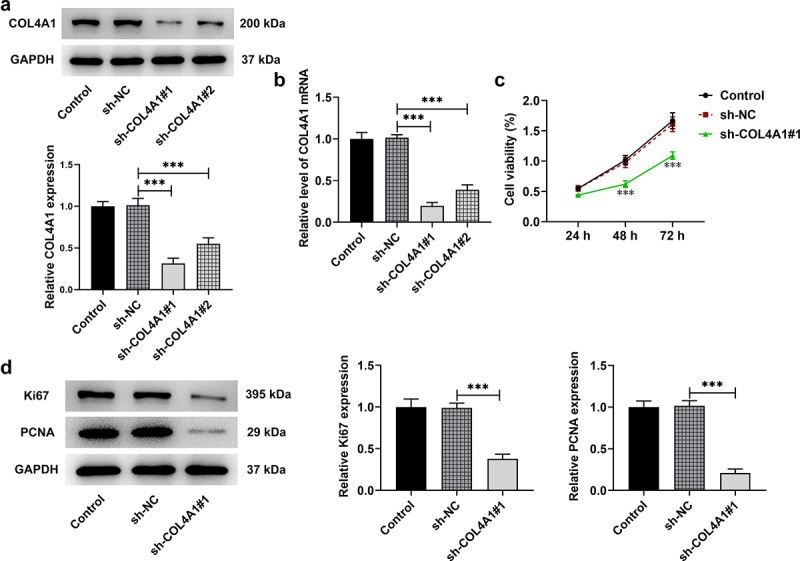
COL4A1 insufficiency abrogated MKN-45 cell proliferation. (a,b) The interference efficiency of COL4A1 was examined by RT-qPCR and Western blot, respectively. (c) The cell viability was detected using CCK-8. (d) The expressions of proliferation-related proteins were detected using Western blot.

In order to knock down COL4A1 expression, short hairpin RNA targeting COL4A1 was employed to transfect MKN-45 cells, and the transfection efficacy was detected. In comparison with sh-NC, the mRNA and protein expressions of COL4A1 were distinctly decreased after transfection with sh-COL4A1 plasmids. Notably, sh-COL4A1#1 had better transfection efficacy, as evidenced by lower expression of COL4A1 in contrast with that in sh-COL4A1#2 group (Figure 2a-b). Therefore, sh-COL4A1#1 was adopted for subsequent experiments.

With the aim of investigating the effects of COL4A1 silence on cell proliferation, CCK-8 was applied. In comparison with sh-NC, the proliferation of MKN-45 cells was greatly decreased after depleting COL4A1 expression (Figure 2c). Likewise, results obtained from Western blot revealed that the protein levels of Ki67 and PCNA, which are deemed proliferation indicators, were significantly downregulated in COL4A1-silenced MKN-45 cells (Figure 2d). The above-mentioned results indicated that COL4A1 silence helped to inhibit the proliferation of GC cells.

## COL4A1 silence inhibited the invasion, migration and EMT of MKN-45 cells

**Figure 3.**
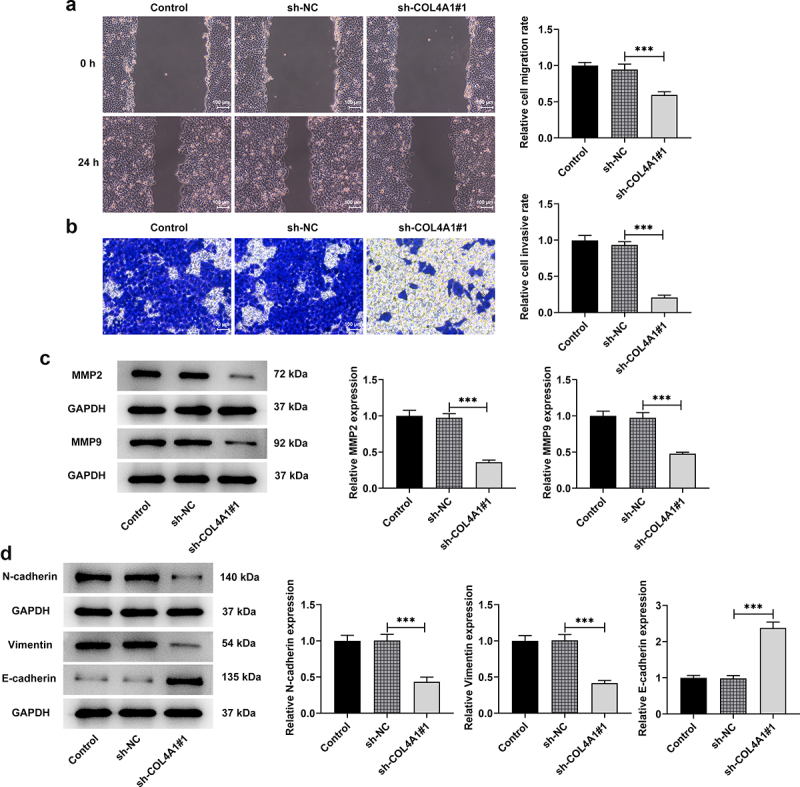
COL4A1 shortage inhibited the invasion, migration and EMT of MKN-45 cells. Cell migration and invasion were respectively evaluated by (a) wound healing and (b) transwell assays. (c) Western blot analyzed the protein levels of metastasis-related factors. (d) The expressions of EMT-related proteins were detected using Western blot.

To explore the effects of COL4A1 silence on the invasion, migration and EMT of MKN-45 cells, transwell and wound healing assays were performed, and related protein expressions were detected. As Figure 3a depicts, the relative cell migration rate was greatly decreased after transfection with sh-COL4A1#1 in contrast with sh-NC, revealing that COL4A1 silence exhibited inhibitory effects on cell migration. Similarly, the relative cell invasive rate was also decreased in COL4A1-silenced MKN-45 cells (Figure 3b). Besides, the downregulated expressions of MMP2 and MMP9 were also observed after COL4A1 was silenced (Figure 3c). Moreover, COL4A1 silence raised E-cadherin expression but cut down N-cadherin and vimentin expressions in comparison with the sh-NC group, suggesting the suppressive effects of COL4A1 silence on EMT (Figure 3d). To sum up, COL4A1 silence could impede the invasion, migration and EMT of GC cells.

## COL4A1 modulated the Hedgehog signaling pathway

**Figure 4.**
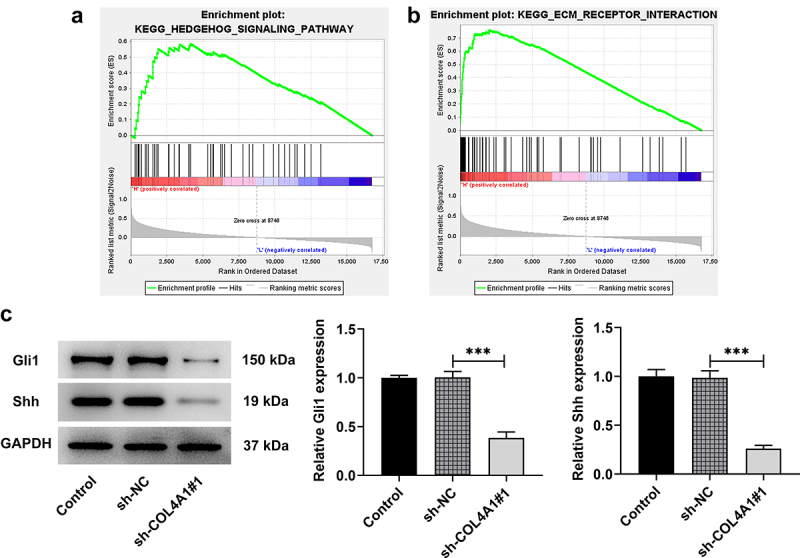
COL4A1 modulated the Hedgehog signaling pathway. (a) GSEA detected the association between COL4A1 and Hedgehog signaling. (b) Western blot analyzed the protein levels of related factors in Hedgehog signaling.

Based on the data on Gene Set Enrichment Analysis (http://www.gsea-msigdb.org/gsea/index.jsp), we found that COL4A1 was involved in the regulation of Hedgehog signaling pathway (Figure 4a). To further confirm this finding, Western blot was applied to detect the expressions of Hedgehog signaling pathway-related proteins, including Gli1, Shh and PTCH. As Figure 4b displays, COL4A1 silence upregulated PTCH expression but downregulated the expressions of Gli1 and Shh in contrast with sh-NC group, revealing that COL4A1 participated in the regulation of Hedgehog signaling pathway-related proteins.

## COL4A1 silence suppressed the malignant properties of MKN-45 cells via blocking Hedgehog signaling pathway

**Figure 5.**
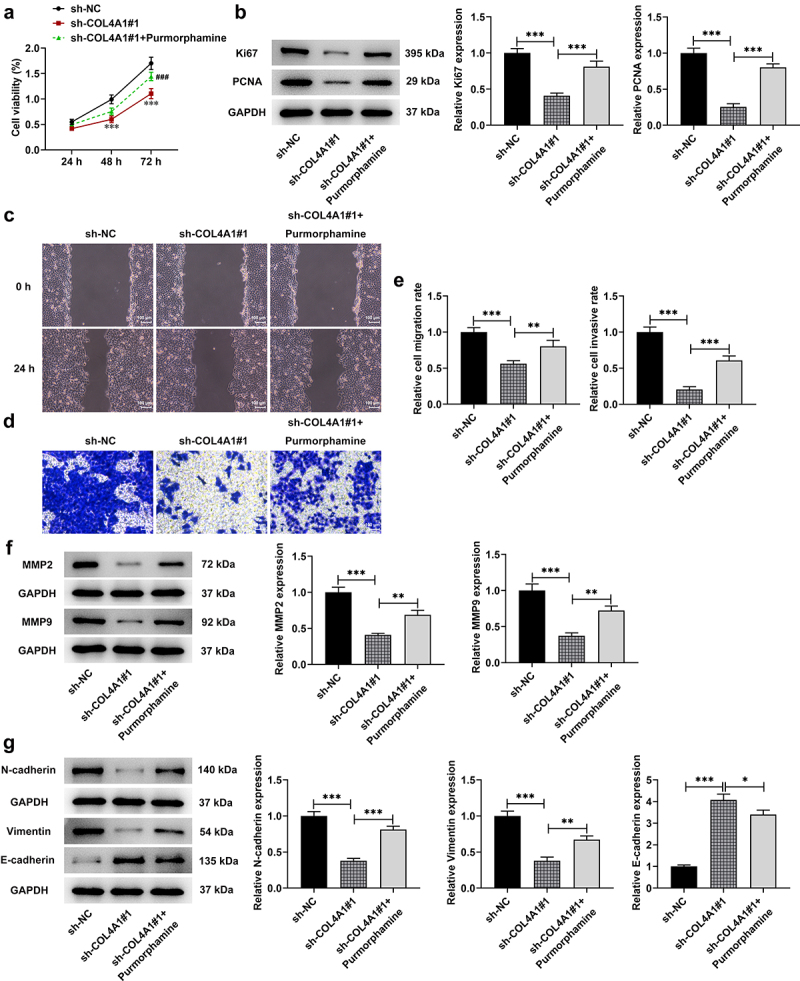
COL4A1 silence suppressed the malignant properties of MKN-45 cells via blocking Hedgehog signaling pathway. (a) The cell viability was detected using CCK-8. (b) Western blot tested Ki67 and PCNA protein levels. Cell migration and invasion were respectively evaluated by (c) wound healing and (d) transwell assays. (e) Western blot analyzed the protein levels of metastasis-related factors. (f) The expressions of EMT-related proteins were detected using Western blot.

To explore the effects of COL4A1 silence on Hedgehog signaling pathway, purmorphamine, an agonist of Hedgehog signaling pathway, was employed to treat MKN-45 cells for 48 h. Then, a series of cellular biological experiments were conducted. According to the results obtained from CCK-8 assay, the decreased proliferative capacity on account of COL4A1 silence was then revived by purmorphamine treatment (Figure 5a). Besides, relative to sh-NC, COL4A1 interference notably cut down Ki67 and PCNA expressions; however, the decreased expressions of these proteins were then increased by purmorphamine (Figure 5b).

Compared with sh-NC, the relative migration rate was greatly decreased in COL4A1-silenced MKN-45 cells, which was then increased after purmorphamine treatment (Figure 5c). Similarly, the inhibitory effects of COL4A1 silence on the relative cell invasive rate were then partially abolished by purmorphamine, as evidenced by the increased invasive rate in sh-COL4A1#1 + purmorphamine group (Figure 5d). Additionally, the expressions of MMP2 and MMP9 were partially enhanced by purmorphamine in comparison with that in sh-COL4A1#1 group (Figure 5e). Moreover, the influences of COL4A1 silence on E-cadherin, N-cadherin and vimentin expression were then reversed by purmorphamine treatment, as evidenced by the reduced E-cadherin expression and elevated expressions of N-cadherin and vimentin in sh-COL4A1#1 + purmorphamine group (figure 5f). To conclude, the above-mentioned results suggested that COL4A1 silence inhibited the malignant progression of GC via blocking Hedgehog signaling pathway.

## Discussion

This study was the first to probe into the relationship between COL4A1 and Hedgehog signaling pathway in GC as well as the detailed molecular mechanism. For the reliability of the results in the following experiments, the mRNA and protein expressions of COL4A1 in GC cells were detected. Thereafter, a series of cellular biological experiments were conducted to explore the biological role of COL4A1 silence in GC cells. According to GESA, COL4A1 had a close relation with Hedgehog signaling pathway. The detection of Hedgehog signaling-related factors was conducted to validate this conjecture. In order to figure out the detailed mechanism of COL4A1, purmorphamine was used to treat MKN-45 cells.

Through investigation, we discovered that COL4A1 was overexpressed in GC cells and its knockdown inhibited the proliferation of MKN-45 cells. Besides, the migratory, invasive and EMT abilities of MKN-45 cells were all attenuated by the deficiency of COL4A1. Additionally, COL4A1 was determined to contribute to the activity of Hedgehog signaling pathway. Moreover, it was further testified that COL4A1 silence exhibited its inhibitory effects on gastric cancer cells via blocking Hedgehog signaling pathway.

COL4A1 is a critical member of the basement membrane of many human tissues and cell types [23]. It was reported that COL4A1 serves as a novel oncogene in many kinds of tumors and participates in the process of multiple malignancies. For example, COL4A1 was overexpressed in hepatocellular carcinoma cells, and its upregulation stimulated FAK-Src signaling to boost hepatocellular carcinoma growth and metastasis [24]. Zhang et al. held the opinion that the overexpression of COL4A1 exhibited promotive effects on the progression of liver cancer, while the silence of COL4A1 yielded the oppositive effects [25]. Our experiments demonstrated that COL4A1 gained a huge growth in GC cells. More importantly, EMT was testified to be a key regulator in the migration and invasion of cancers and is responsible for the cancer progression [26]. It was testified that vimentin was a more vital determinant for cancer metastasis [27,28], and study has confirmed that the inhibition of vimentin could suppress the EMT in GC [29]. In the same way, we found that COL4A1 silence downregulated the expressions of Ki67, PCNA, MMP2, MMP9, N-cadherin and Vimentin but promoted E-cadherin expression in MKN-45 cells, revealing that COL4A1 silence could help to suppress the proliferation, metastasis and EMT in gastric cancer.

It is well documented that Hedgehog signaling pathway is linked to cellular events in diverse tumors [30]. Take gastric cancer as an example, Hedgehog signaling pathway contributes to the aggressiveness of gastrointestinal tumors [31]. Hui et al. demonstrated that the inhibition of Hedgehog signaling pathway could dramatically suppress tumor growth and metastatic spread in triple-negative breast cancer [32]. Obviously, the block of Hedgehog signaling pathway might be a novel treatment for cancers.

According to GSEA, COL4A1 is related to the regulation of Hedgehog signaling pathway. PTCH1, PTCH2 and GLI1 are the dominant target genes of Hedgehog signaling pathway [33]. Besides, a previous study has verified that Gli1, which serves as a transcriptional activator, induced gene expression and was viewed to be a biomarker of pathway activation [34]. In this study, it was discovered that COL4A1 silence lessened Gli1 expression in MKN-45 cells, revealing that COL4A1 silence could function as an inactivator of Hedgehog signaling pathway. Further, it was also found that COL4A1 silence exhibited suppressive effects on the malignant phenotypes of GC cells via blocking Hedgehog signaling pathway.

## Conclusion

This study investigated the detailed molecular mechanism of COL4A1 in GC and identified COL4A1 as a valuable molecular marker for the treatment of GC therapy, providing a new angle for the study of GC.
